# Preclinical curriculum of prospective case-based teaching with faculty- and student-blinded approach

**DOI:** 10.1186/s12909-019-1453-x

**Published:** 2019-01-23

**Authors:** Sarah Waliany, Wendy Caceres, Sylvia Bereknyei Merrell, Sonoo Thadaney, Noelle Johnstone, Lars Osterberg

**Affiliations:** 1 Department of Medicine, Stanford University School of Medicine, 1265 Welch Road, MSOB x152, Stanford, CA 94305 USA; 2 Department of Surgery, Stanford University School of Medicine, Stanford, CA USA; 3 Program for Bedside Medicine, Stanford University School of Medicine, Stanford, CA USA; 4 Department of Pediatrics, Stanford University School of Medicine, Stanford, CA USA

**Keywords:** Case-based teaching, Preclinical curriculum, Hindsight bias, Real-world cases, Prospective case-based discussion

## Abstract

**Background:**

Case-based teaching with real patient cases provides benefit of simulating real-world cognition. However, while clinical practice involves a prospective approach to cases, preclinical instruction typically involves full disclosure of case content to faculty, introducing hindsight bias into faculty teaching in medical curricula.

**Methods:**

During 2015–2018, we piloted an optional medical school curriculum involving 6–7 one-hour sessions over a 3-month period each year. New groups enrolled each year from first- and second-year classes. A facilitator provided a blinded physician discussant and blinded students with case information during and not in advance of each session, allowing prospective case-based discussions. Cases were based on real patients treated in the Department of Medicine. Clinical material was presented in the chronologic sequence encountered by treating physicians. Content covered a median of 5 patient visits/case (range: 2–10) spanning over months. A 14-item survey addressing components of the reporter-interpreter-manager-educator (RIME) scheme was developed and used to compare self-reported clinical skills between course participants and non-participant controls during the 2016 course iteration.

**Results:**

This elective curriculum at Stanford School of Medicine involved 170 preclinical students (22.7% of 750 eligible). During the 2016 course iteration, a quasi-experimental study compared self-reported clinical skills between 29 course participants (response rate: 29/49 [59.2%]) and 35 non-participant controls (response rate: 35/132 [26.5%]); students self-assessed clinical skills via the RIME-based survey developed for the course. Two-sample *t*-tests compared the change in pre- and post-course skills between course participants and non-participants. Of 15 Department of Medicine faculty members invited as discussants, 12 (80%) consented to participate. Compared with controls, first-year participants self-assessed significantly greater improvement in understanding how clinicians reason through cases step-by-step to arrive at diagnoses (*P* = 0.049), work through cases in longitudinal settings (*P* = 0.049), and share information with patients (*P* = 0.047). Compared with controls, second-year participants self-assessed significantly greater improvement (*P =* 0.040) in understanding how clinicians reason through cases step-by-step to arrive at diagnoses.

**Conclusions:**

Prospective case-based discussions with blinding of faculty and students to clinical content circumvents hindsight bias and may impart real-world cognitive skills as determined by student self-report.

## Background

In the medical school curriculum, preclinical medical education has shifted over the past few decades from a focus on traditional discipline-specific teaching (eg, physiology, anatomy, pharmacology) towards case-based learning (CBL) [1, 2]. Case-based learning in the preclinical curriculum provides a central benefit of imparting clinical problem-solving skills at an early stage of medical training. Broader research on cognitive load theory in adults has suggested that learning is most effective when individuals are asked to apply knowledge and skills to real-life scenarios [3, 4]. Accordingly, both thought leaders and researchers in medical education have advocated for the use of actual real-world clinical cases in CBL [5–9]. Using authentic patient cases in CBL allows curricular case-based discussions to more closely emulate the complexity of real-world clinical cognition, including exposing students to ambiguous or conflicting clinical data and to unexpected factors that impact clinical care (e.g., personality differences between patients and providers, loss to follow-up) [5, 6]. Furthermore, multiple studies have demonstrated that utilization of cases in curricula is effective in increasing basic science and clinical knowledge of medical students and enhancing their skills in applying that knowledge towards patient cases [10–12].

One of the discrepancies between cognitive skills applied in real-world clinical practice versus those illustrated through the contemporary CBL format is the presence of hindsight bias in the latter, especially pertaining to instruction by faculty. Hindsight bias occurs when those who know the correct diagnosis overestimate the likelihood that they would have been able to determine the diagnosis had they been asked to do so in a prospective setting [13–19]. Although the specific logistics of CBL are variable between different institutions, one common feature of CBL reported in the literature that also applies to our institution’s preclinical curriculum is the full disclosure of case information to the faculty that facilitate CBL sessions [20–22]. As groups of students work through cases, faculty typically facilitate the discussion, especially by guiding students to discuss particular topics and potentially navigating students if they appear to be leading towards the incorrect diagnosis [20–24]. By granting faculty the benefit of knowing the full content of the case, the traditional CBL format introduces hindsight bias, which carries the risk of preventing preclinical students from being exposed to the errors or uncertainties that can occur when considering between competing diagnostic possibilities in the setting of ambiguous clinical data [13–16, 25]. In the clinical setting, the nature of patient care compels both attendings and trainees to approach cases prospectively without the benefit of knowing how the case will unfold in advance. During clinical rotations, medical students benefit from this real-world setting as they learn from their team’s approach to clinical scenarios in the absence of hindsight bias. Through a retrospective view of cases by preclinical instructors, CBL creates a discrepancy between preclinical and clinical education and – more notably – between preclinical education and real-world clinical practice.

One of the earlier case-based didactic methods used before the current format of CBL was the clinicopathologic conference (CPC) [26–30]. Massachusetts General Hospital introduced CPCs in 1910 with the objective of replacing topic-based lectures with case-based discussions [26]. Although other institutions later implemented their own variations of CPCs, the original intended format of CPCs involved physician discussants working through cases that they had not seen in advance of the conferences in front of a large audience of attendings and trainees; case information was provided to discussants in real time during CPCs by presenters with access to the cases. Through the use of blinded discussants, CPCs allowed the audience to learn from the clinical cognition of the discussant in the absence of hindsight bias. Clinicopathologic conferences have become less utilized over time, with stated reasons including the pressure on the discussant of arriving at the correct diagnosis, the excessive focus on the final diagnosis and disease process, and the neglect to consider other factors pertinent to the case, including psychological and social factors [29]. This decline of CPCs has led to the loss of the benefit of prospective clinical cognition by a blinded faculty discussant in medical education.

At our institution, we piloted an optional curriculum from 2015 to 2018 that aimed to expose preclinical students to prospective real-world clinical reasoning by integrating components of both more contemporary CBL methods and the less commonly used CPC format. During course sessions, an unblinded facilitator provided a physician discussant and a group of students with case information *during* and not in advance of course sessions, allowing both the discussant and students to be blinded to the cases and to reason through clinical material in the absence of hindsight bias. While traditional case-based teaching methods involve small group sessions (ranging from 1 to 30 students, and on average involving 2 to 15 students per group) [2] and traditional CPC sessions involve larger audiences of both trainees and attendings [26], we piloted this course with intermediate-sized groups of 45 to 59 students per year, including both first- and second-year students. All curricular cases were based on real patients treated in the outpatient setting at the Department of Medicine at our institution, and the facilitator presented case information in the chronologic sequence encountered by the treating physician. This format allowed the case-based discussion to closely emulate a real-life clinical scenario. Furthermore, we integrated topics in not only interpretation (e.g., deliberating a differential diagnosis) but also management, patient education, and the impact of socioeconomic factors on patient care (when relevant) into each case discussion. We also conducted a prospective quasi-experimental study to compare self-reported clinical skills between course participants and non-participants.

## Methods

### Setting and participants

The Primary Care Presentations course was offered to both first- and second-year medical students at our institution, Stanford University School of Medicine. From 2015 to 2018, out of 750 preclinical students eligible to participate in the course, 170 (22.7%) participated in the course, including 99 first-year and 71 second-year medical students. New groups of students participated in the course each year from the cohorts of first- and second-year medical students. We conducted a prospective quasi-experimental study design comparing self-reported clinical skills between participants of the 2016 course iteration and a control convenience sample of medical student respondents of the same academic years at the same institution that did not participate in the curriculum. The course participants and the control students participated in the same required medical school curricula throughout both the first and second preclinical years, with the reported elective course being the only difference in curricular experience between the two groups.

### Curriculum design

The course was offered on a quarterly basis (3-month period each year) and consisted of 27 one-hour sessions from 2015 to 2018, with 6–7 sessions held during each annual quarter. Case presentations developed for course sessions were based on patients treated in the Department of Medicine at Stanford Health Care. Cases were chosen that would allow not only interpretation of reported history, physical exam, and diagnostic data but also discussion of management steps and patient education opportunities. Clinical information was retrieved through electronic medical records (EMR) and compiled into a presentation format (using Microsoft PowerPoint) by one author (WC) and edited by two other authors (SW and LO). The case material was organized in the chronologic sequence encountered by the treating primary care physician (PCP), as recommended in the literature on case-based teaching [5]. Clinical content included information 1) available in the EMR prior to the first visit, 2) gathered over that initial consultation, and 3) from subsequent visits to the PCP and other health care professionals. Content for each visit comprised relevant data available in the EMR, including history, physical exam findings, lab values, other diagnostic results, treatment plan, and patient education steps pursued by the treating clinicians. Patient identifiers such as names, medical record numbers, and dates of birth were not provided in presentations.

Examples of diagnoses covered by the course cases included the following: alcoholic cirrhosis with hepatic encephalopathy; polycystic kidney disease with secondary hypertension; complications associated with type 2 diabetes mellitus in the setting of medication noncompliance; and cardiovascular disease after Hodgkin’s lymphoma treatment. Presentations included content over a median of 5 patient visits (range: 2–10) spanning over multiple months to incorporate instruction in longitudinal patient care. Many cases involved discussions in managing chronic conditions such as diabetes, heart disease, hypertension, chronic kidney disease, and long-term complications of cancer treatment. Longitudinal management topics included medication regimen changes for diabetes, post-splenectomy vaccinations, managing neuropathic pain, and others. Longitudinal patient education topics included weight loss counseling and addressing medication nonadherence.

**Fig. 1:**
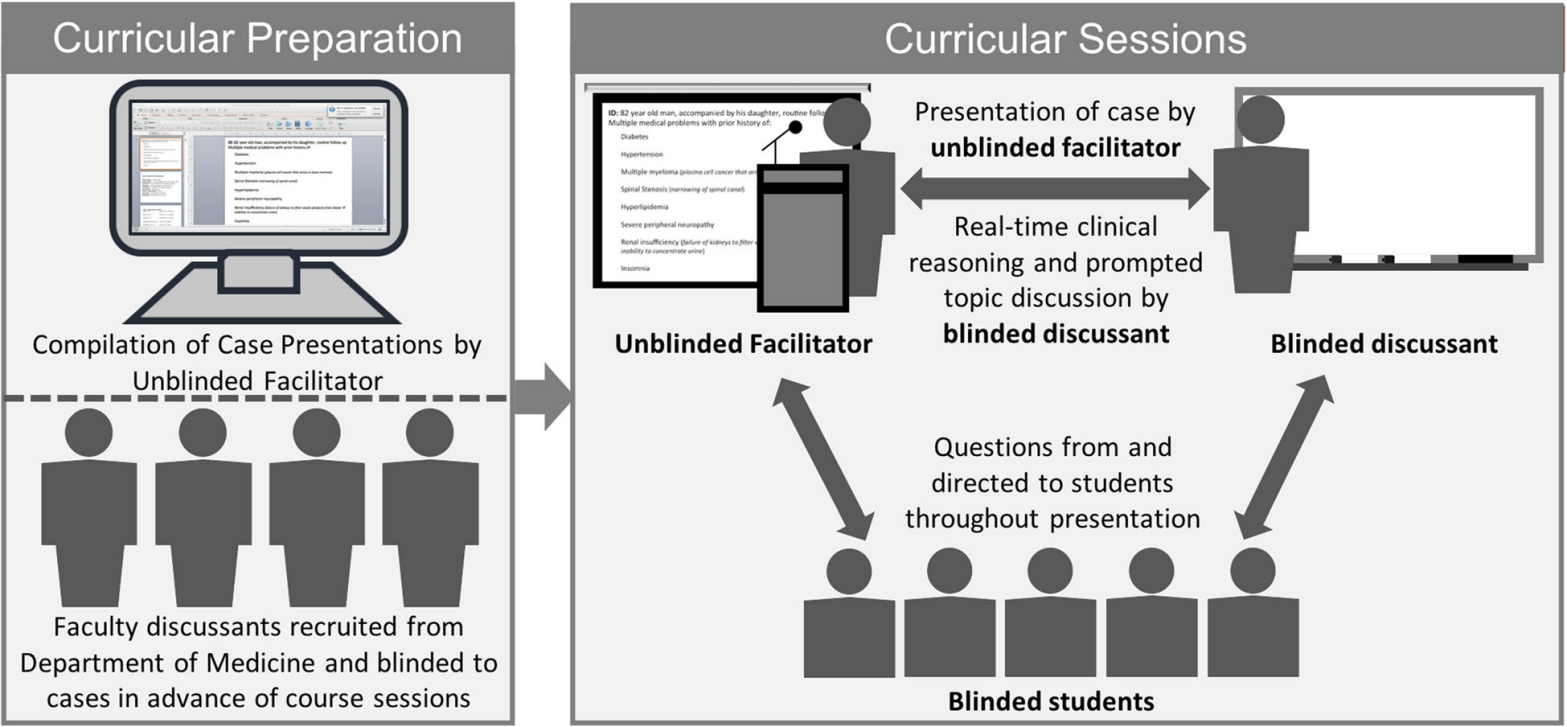
Faculty- and student-blinded prospective case-based teaching method

Faculty were recruited from the Department of Medicine of Stanford School of Medicine to participate as discussants in the course sessions. Out of 15 attendings (ranging from chief residents to professors of medicine) invited to participate as discussants, 12 (80%) accepted the invitations; out of the three that declined, two cited scheduling conflicts and one cited reluctance to participate as a blinded discussant. During each course session, a prepared physician facilitator (WC or LO) presented a case to a discussant and to student participants; both the discussant and students were blinded to the case before the session, including the eventual diagnosis. The facilitator prompted the discussant to explain his/her interpretation of each set of information while moving sequentially through the case (see Fig. 1), role-modeling the spontaneous “think aloud” clinical cognition of the discussants [31, 32]. The facilitator and discussants also asked interactive questions to consistently engage students in the case-based discussion throughout the presentation. The facilitator had prepared specific prompting questions to ask students to ensure student participation and engagement in the case-based discussions. Discussants recruited for the course were also instructed in advance to consistently ask questions to engage students in the process of working through the cases.

The case presentation format allowed the case to unfold according to how the patient had presented in the actual clinical setting while students learned from the blinded, unrehearsed discussant’s clinical cognition. Clinical skills that discussants were prompted to demonstrate included 1) interpretation of history, physical exam findings, and diagnostic results, 2) formulation and iterative reevaluation of problem lists and differential diagnoses, 3) recommendations for short- and long-term therapeutics and management, and 4) opportunities for patient education (see examples of case topics in Table 1). Depending on the case, the course facilitator also prompted discussants to describe how to approach social and psychological factors that could be pertinent to the care of that patient, such as providing motivational interviewing for weight loss or counseling patients with a fear of going to the Emergency Department. Furthermore, when relevant, the facilitator and discussant acknowledged ambiguities or uncertainties in interpreting certain diagnostic data, providing a more realistic illustration of the diagnostic process and placing less emphasis on simply reaching the correct diagnosis. After the discussant had explained his/her treatment recommendations and potential opportunities for patient education and information sharing, the facilitator discussed the actual treatments (and educational topics if documented in clinical notes) that had been pursued by the patient’s treating physician.

**Table 1:** Examples of cases used in curriculum with topics in interpretation, short- and long-term management, and patient education

Age/Sex	Diagnosis	Interpretation	Management	Patient Education	# of patient visits
57 / F	Recurrent pancreatitis and erythema nodosum	• Differential diagnosis for 1) left upper quadrant abdominal pain, 2) erythema nodosum, and 3) recurrent pancreatitis • Bedside index of severity in acute pancreatitis (BISAP) score	• Cornerstones of pancreatitis management in inpatient & outpatient setting	• Counseling patients with fear of going to Emergency Department	3
82 / M	Serotonin syndrome secondary to polypharmacy in the elderly	• Medication interactions in setting of serotonin syndrome • Physical exam findings in serotonin syndrome • Differential diagnosis for 1) delirium and 2) myalgia	• Consolidating medications • Home safety evaluation • Diabetes control • Primary prevention of myocardial infarction • Managing neuropathic pain and myalgia	• Teaching self-monitoring of blood glucose • Substance use counseling • Educating family caregivers	2
49 / M	Secondary gout & cardiovascular disease in Hodgkin’s Lymphoma survivor	• Long-term complications in survivors of Hodgkin’s Lymphoma after treatment • Differential diagnosis for 1) foot pain and 2) chest pain	• Management of gout • Post-splenectomy vaccinations • Cardiology & nutrition referrals • Managing aortic stenosis after identifying cause	• Dietary counseling for gout • Weight loss counseling	8
55 / F	Type 2 Diabetes	• Criteria for diagnosing diabetes • Changes in hemoglobin A1C and lipid panel in the setting of weight change	• Dietary recommendations to promote weight loss • Diabetes management • Referrals to nutrition, sleep clinic, and psychology • Managing care for patients who irregularly follow-up	• Lifestyle counseling and motivational interviewing for weight loss • Reviewing food questionnaire results	8

### Assessment methods

The course curriculum was evaluated in two ways: 1) a quasi-experimental pre- and post-intervention study comparing self-reported clinical skills of course participants and a control sample of non-participants and 2) evaluation of the course by student course participants.

### Survey of self-reported clinical skills

We developed a 14-item survey administered electronically to assess students’ self-reported clinical skills in the following domains: reporter (2 items), interpreter (4 items), manager (3 items), educator (1 item), and other skills (4 items) [33]. The reporter-interpreter-manager-educator (RIME) scheme was used to structure the survey since clinical students at our institution are evaluated on clerkship performance by a RIME-based assessment method [34]. A 5-point Likert scale was used for all items, with 1 indicating “Strong Disagreement” and 5 indicating “Strong Agreement” with the statement. The Stanford School of Medicine Institutional Review Board determined that this study did not meet the federal definition of human subject research and certified this study as exempt from review.

All participants of the 2016 course sessions were invited to complete the survey within 2 weeks before the first session and within 2 weeks after the last session. During the same time frames, the survey was also administered to all first- and second-year non-participants of the course with respondents serving as the control group. Surveys were submitted anonymously by course participants and the control group. Students were blinded to the comparison being made between the two groups to avoid biasing the results.

### Student evaluation of curriculum

Student participants submitted anonymous evaluations of the curriculum from 2015 to 2018. Students rated the “overall quality of course” on a 5-point Likert-type scale (1 = Poor to 5 = Excellent). Students were also given the option to anonymously submit qualitative written comments regarding skills or knowledge that the course provided them and suggestions for improvement.

### Statistical analysis

For the survey on self-reported clinical skills, students’ post-intervention survey responses were matched to pre-intervention responses, and the change in the scores (hereafter referred to as “score change”) was calculated as post-intervention minus pre-intervention score for each of the 14 items for each respondent, with a positive score change indicating an improvement in self-reported clinical skills after the course. Two-sample student’s *t*-tests were used to compare the score change for each item between course participants and the control group, setting significance at *P* < 0.05. To determine the magnitude of differences between both groups, associated effect sizes were calculated using Cohen's d, with thresholds of 0.2, 0.5, and 0.8 being used to define small, medium, and large effect sizes, respectively [35]. Statistical analyses were performed using Statistical Analysis Systems, version 9.4 (SAS Institute, Inc., Cary, NC).

## Results

### Self-reported clinical skills

**Fig. 2:**
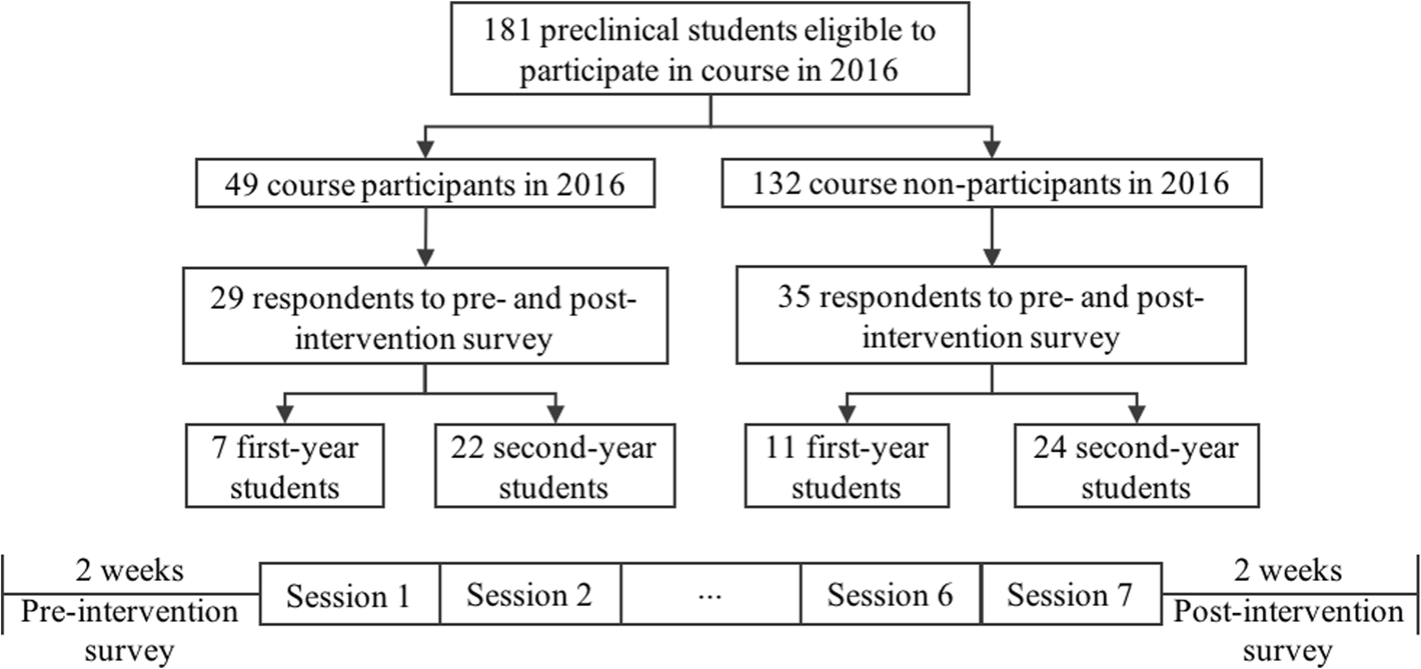
Consort diagram of quasi-experimental study comparing change in self-reported clinical skills between course participants and a control convenience sample of non-participants

Out of the 49 course participants in 2016, both pre- and post-intervention surveys were completed by 29 (59.2%) participants, including 7 first-year and 22 second-year students, for academic year-specific response rates of 30.4% (7/23) among first-year students and 84.6% (22/26) among second-year students (see Fig. 2). The 29 course participants that responded to both pre- and post-intervention surveys are hereafter referred to as the “intervention group.”

The survey was also sent to all course non-participants, with respondents serving as the convenience sample control group. Out of the 132 non-participants that were eligible for the course, 35 (26.5%) responded to both pre- and post-intervention surveys, including 11 first-year and 24 second-year students, for academic year-specific response rates of 16.4% (11/67) among first-year students and 36.9% (24/65) among second-year students. The 35 non-participants of the course that responded to both pre- and post-intervention surveys are hereafter referred to as the “control group.”

**Fig. 3:**
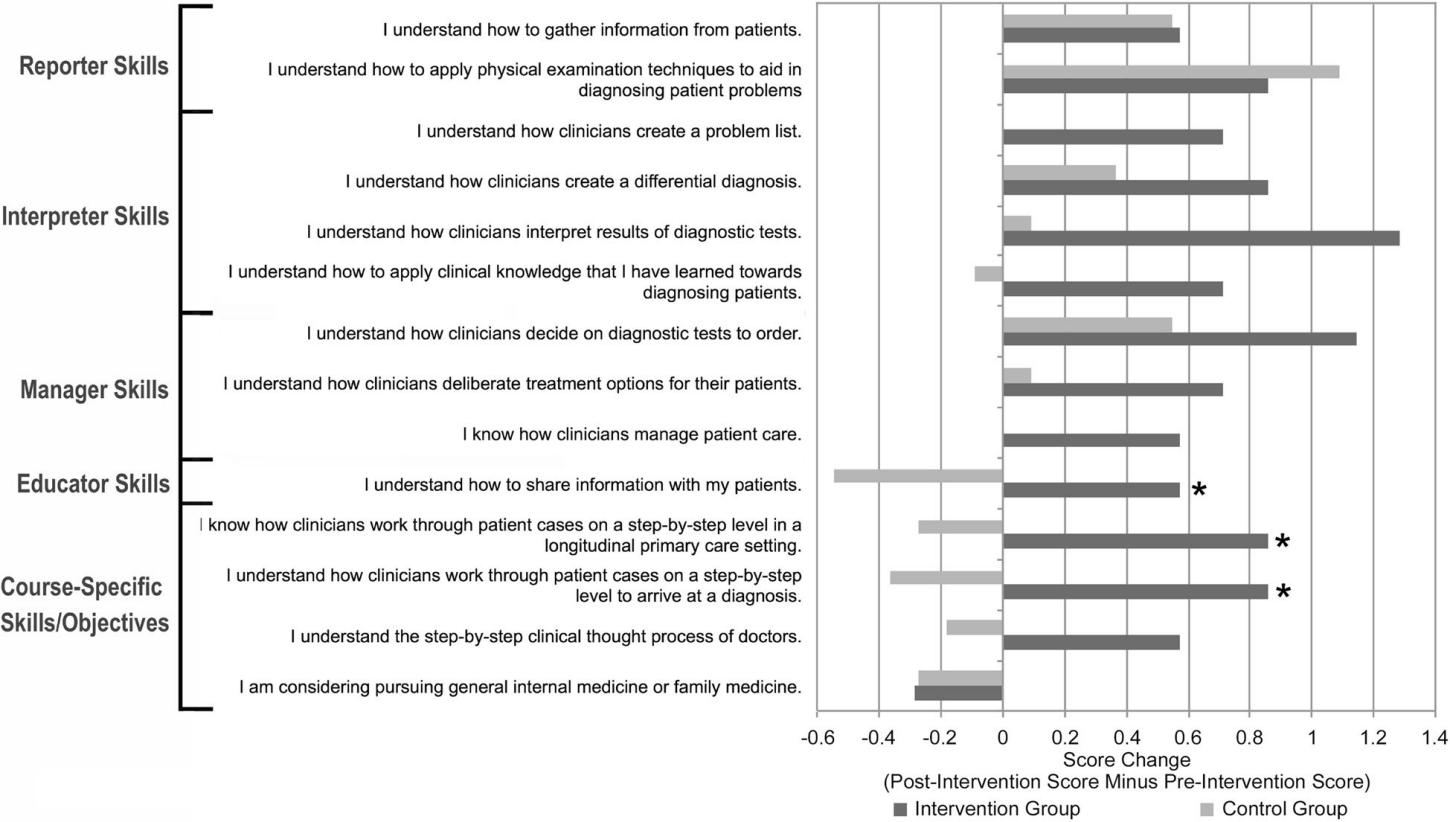
Comparison of mean score change (post-course score minus pre-course score) between participants and non-participants among first-year medical students. * for *p* < 0.05 and d > 1.0

Among first-year respondents, the intervention group (*n* = 7) had a more positive average score change compared with the control group (*n* = 11) for 12 out of 14 items across all survey domains (see Fig. 3), including reporting (1 out of 2 items), interpretation (4/4), management (3/3), patient education (1/1), and other skills (3/4). The intervention group reported a significantly more positive score change in the following three items:

- *I understand how clinicians work through patient cases on a step-by-step level to arrive at a diagnosis* (*P* = 0.049, d = 1.04)
- *I know how clinicians work through patient cases on a step-by-step level in a longitudinal primary care setting* (*P* = 0.049, d = 1.02)
- *I understand how to share information with my patients* (*P* = 0.047, d = 1.08)

**Fig. 4:**
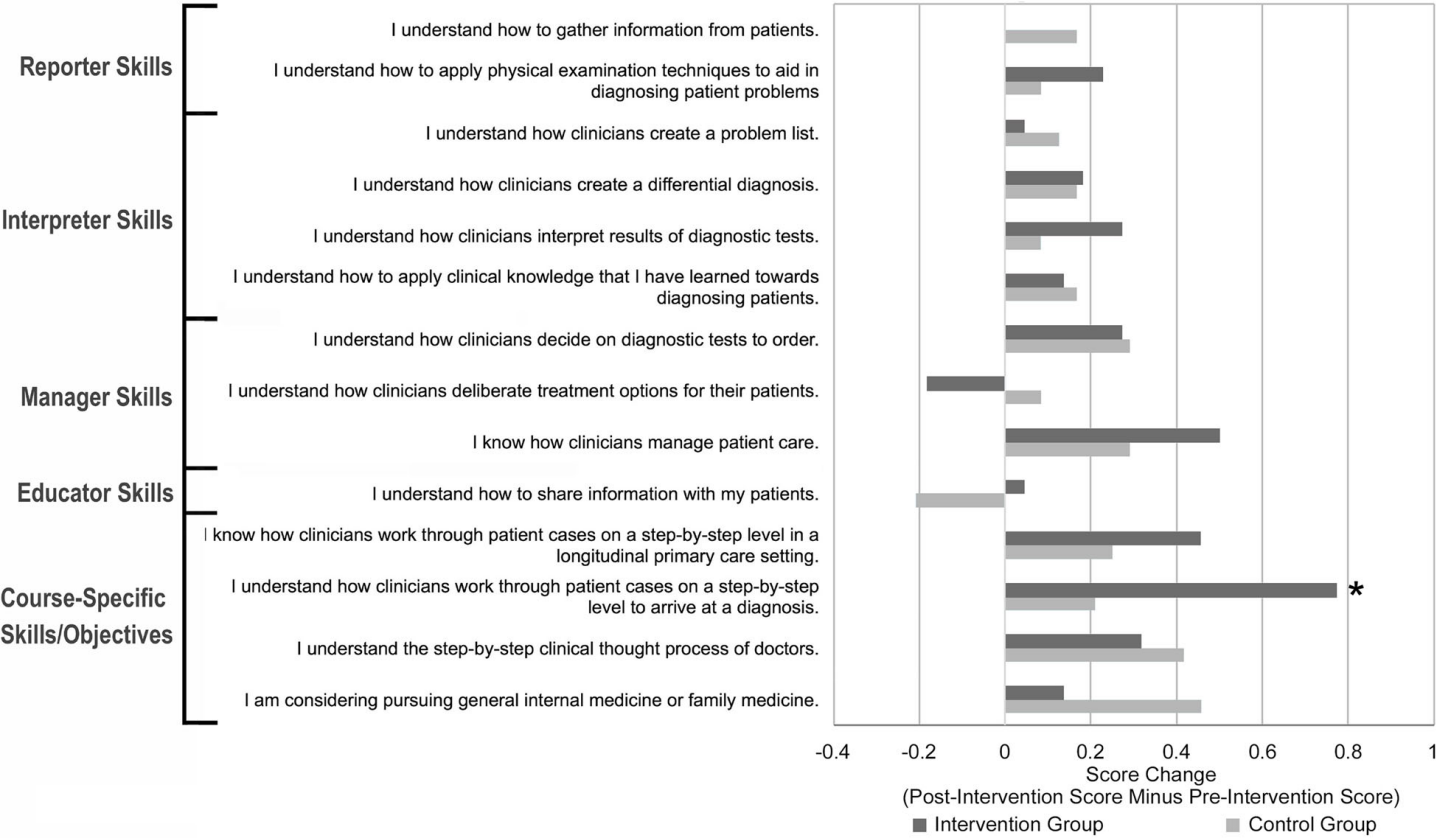
Comparison of mean score change (post-course score minus pre-course score) between participants and non-participants among second-year medical students. * for *p* < 0.05 and d > 1.0

Among second-year respondents (see Fig. 4), the intervention group (*n* = 22) had a more positive score change compared with the control group (*n* = 24) for 7 out of 14 items across all domains, including reporting (1 out of 2 items), interpretation (2/4), management (1/3), patient education (1/1), and course-specific objectives (2/4). The intervention group reported a significantly more positive score change (*P* = 0.04, d = 0.66) in the following item: *I understand how clinicians work through patient cases on a step-by-step level to arrive at a diagnosis*.

### Course evaluations

Anonymous course evaluations were submitted by 62 (36.5%) out of 170 participants (2015: *n* = 16 [response rate: 35.6%]; 2016: *n* = 16 [32.7%]; 2017: *n* = 20 [40.8%]; 2018: *n* = 10 [37.0%]). The mean (standard deviation) ratings for “overall quality of course” were 4.69 (0.46) in 2015; 4.50 (0.52) in 2016; 4.50 (0.60) in 2017; and 4.80 (0.40) in 2018. Since qualitative assessments were submitted as mostly brief comments by only 2 to 7 students each year from 2015 to 2018, a thematic analysis of the comments was not done. Instead, all comments submitted by students have been included in Table 2.

**Table 2:** Qualitative assessment of course submitted by student participants (*n*=19)

	What are your comments on the course overall?	What are your suggestions for improvement?
2015	• “Very interesting course. It is a valuable experience to be exposed to how attendings think about cases as they are presented with new information. I hope that this course will be given every year in the future.” • “This course was very well taught and helped outline, in a meaningful way, how physicians work through cases to arrive at a diagnosis.” • “Great interactive style.” • “ability to apply what we learn and also see the thought process doctors go through in terms of their mental organizations”	• “More case variety”
2016	• “This course will help frame your medical education and help you think of a differential.” • “The Primary Care seminar series was an engaging way to learn a broad set of clinical reasoning skills.” • “This was a fantastic opportunity to get a primer on clinical reasoning for a first-year student.” • “If you are interested in learning more about the clinical reasoning from actual patient cases this is really a great opportunity to do it!” • “I learned how to develop a differential diagnosis, how to think like a primary care clinician, how to apply knowledge in a clinical setting.” • “The clinical reasoning throughout this seminar helped me really understand the applications of the physical exam concepts that I learned this quarter and was a great experience to learn from clinical experts and how they reason.”	No suggestions were given.
2017	• “Wonderful course that is a fantastic introduction to differential diagnosis. This is a fun seminar that will introduce you to a variety of primary care fields.” • “Good class to take to learn how to think through clinical cases.”	No suggestions were given.
2018	• “Great class for understand the differentials process” • “This course is a good way to gain exposure to a practicing physician's clinical/diagnostic thought process with messy, real life cases.” • “Learned a lot about physicians' thought processes.” • “I highly recommend this class to all pre-clinical students. I feel like the other courses have to cover so much information that we learn about conditions, but miss the practical information of applying our knowledge to when we see a patient. I think this class was a great example of integrating and applying the information and so critical to our training.” • “It is a good idea to take this course before you head off to clerkships.” • “Learned about how to formulate differentials, problem lists, etc.” • “Really enjoyed this class because got an inside look at how to problem solve patient cases”	No suggestions were given.

## Discussion

Case-based learning (CBL) is a widespread pedagogical method in medical school curricula. Case-based teaching methods have been demonstrated to lead to improved satisfaction of health professional students with their clinical education [12, 36, 37]. Furthermore, integrating cases into curricula has been shown to enhance basic science or clinical knowledge as evaluated by self-assessment surveys [12, 38–40] or objective measures of skills [10–12]. Since adult learning theory suggests that learning is most effective when individuals are asked to apply the newly acquired knowledge to real-life scenarios [3, 4], thought leaders in medical education have encouraged the use of real clinical cases in CBL, allowing curricular teaching to emulate authentic clinical cognition [5, 6]. However, a major deficit to the current format of case-based teaching in the medical school curriculum is the presence of hindsight bias in the setting of full disclosure of case information to faculty instructors. This feature of case-based instruction creates a discrepancy between the clinical reasoning that students are exposed to in the preclinical curriculum and the prospective clinical cognition inherent in real-world medical care. To avoid hindsight bias in preclinical instruction, we developed a curriculum that implemented blinding of both faculty discussants and students during case-based discussions.

Earlier research done to understand cognitive processes of clinicians was largely based on analyses of transcripts of physicians thinking aloud while working through real clinical cases, either through simulated patient encounters [41] or through a format similar to our course, with clinical content provided to physician subjects by researchers in the chronologic sequence encountered by the treating physician [25]. These studies provided insight into nuanced, iterative clinical reasoning skills that clinicians use to generate and evaluate diagnostic hypotheses and to deliberate between management options [25, 41–44]. Findings from these studies guided pioneers in medical education in proposing how to teach clinical problem solving skills [25, 43, 44]. Our curricular format of physician instructors working prospectively through cases without advance knowledge of the cases similarly allows students to benefit from the “think aloud” thought process of clinicians, exposing them to potentially more nuanced clinical reasoning methods. By prompting faculty to discuss their reasoning prospectively while being presented with case information in the sequence originally encountered by the treating physicians, the course role modeled for students the step-by-step process of working through cases to arrive at a diagnosis, leading to significantly greater confidence among course participants compared with non-participants in working through cases in a step-by-step manner.

The predominant preclinical pedagogical format of using curricular cases known to instructors in advance of teaching sessions is limited by the phenomenon of hindsight bias [13, 18]. This bias can occur when instructors are aware of the final diagnosis and overestimate the likelihood that they would have arrived at that diagnosis had they been asked to predict it beforehand. Previous studies have demonstrated hindsight bias among physicians. For example, Arkes et al. [17] divided 75 practicing physicians into five groups and provided each group with the same case history. The “foresight group” was given four possible diagnoses and asked to estimate the probability of each of the diagnoses. The four “hindsight groups” were given the same four options, but each was told in advance that one of those four diagnoses was correct. Compared with the foresight physicians, the hindsight groups assigned significantly greater probability estimates to the diagnoses that they were told were correct.

Another study also demonstrated hindsight bias among 160 physician audience members at four case conferences [18]. Physicians at those conferences were divided randomly into a foresight and hindsight group, with only the latter being informed of the correct diagnosis. After both groups were presented with the clinical information, they were instructed to rank the likelihood of five diagnostic possibilities, with the hindsight group asked to rank the diagnoses the way they would have if they had not already been informed of the final diagnosis. Compared with the foresight group, significantly more members of the hindsight group ranked the correct diagnosis as first (30% vs 50%). The results of those studies suggest that clinical instructors aware of diagnoses in advance of teaching sessions may overestimate the likelihood that they would have reached those same conclusions in real time.

In traditional case-based learning sessions, hindsight bias may skew instruction and facilitation of discussions by faculty, reducing student exposure to the inherent uncertainty that accompanies clinical reasoning when physicians consider between competing diagnostic possibilities in the actual clinical setting. Uncertainty in clinical decision making is an infrequently studied source of distress for medical students and physicians [45–48]. Causes of medical uncertainty include technical sources pertaining to uncertainty about medical information; personal sources relating to obscurity of patients’ wishes; and conceptual sources pertaining to ambiguity of applying guidelines or past experiences to the care of current patients [15]. Past studies have demonstrated a high prevalence of intolerance for uncertainty among medical students, with greater intolerance for uncertainty associated with an aversion to fields such as primary care and psychiatry [47, 48]. Contrary to what might be expected, level of intolerance for ambiguity has been shown not to vary over the 4 years of medical school [48]. Exposure to cases involving ambiguous clinical information is currently not well-integrated in preclinical medical education; the focus on retrospective case-based discussions in the curriculum may lead to overconfidence among medical students in making clinical decisions and less tolerance for navigating diagnostic uncertainty [15, 17, 19]. Our course model of blinded clinicians role modeling their clinical reasoning while working through cases prospectively aimed to increase student exposure to the process of navigating ambiguous clinical presentations. These prospective case-based discussions allowed a more authentic illustration of problem-solving methods in the absence of hindsight bias in a manner more similar to actual clinical cognition.

In addition to the blinding of faculty discussants to the case content, another important distinction between our curriculum and the traditional CBL format is the number of students involved; while CBL as described in the literature usually involves groups of 2 to 15 students per group (ranging up to 30 students) [2], we piloted our curriculum with a group of 40–50 students/year. Compared with CBL, our faculty discussant- and student-blinded teaching format involved more time allocated to role-modeled clinical reasoning by the discussant than to discussion by students. To avoid the sessions from mirroring a lecture format and to promote discussion by students, during all sessions, the course facilitator actively engaged students during the case-based discussions, and discussants were also instructed in advance to involve students in the discussion. Future attempts can be made to implement the blinded-faculty format of this course towards CBL sessions, with an unblinded facilitator providing case information sequentially in “chunks” or through an iterative process to both a blinded discussant and small blinded groups of students; the role of the facilitator would be focused on providing the case information with the discussant’s role being to guide and moderate the case-based discussions. This format of two-way blinding of the faculty instructor and students would allow a prospective approach to cases without risk of hindsight bias impacting the discussion.

In previous decades, many medical schools or training programs used clinicopathologic conferences as a common didactic session [26–30]. The original intended format of clinicopathologic conferences (CPCs) had a similar pedagogical format as our course, with blinded discussants working through cases provided to them by unblinded presenters [26, 27]. A 1994 study on the status of CPCs at academic medical centers found that 80% of surveyed internal medicine residency programs held those conferences, with a primary objective of teaching clinical problem-solving skills and providing a more detailed discussion on a specific topic in internal medicine [26].

The few formal reports that exist describing CPCs suggest that these didactic sessions have become less utilized over time due to excessive focus on the final diagnosis and disease process and the neglect to consider complexities such as social factors pertinent to the cases [29]. Our course implemented a blinded discussant format while imparting not only diagnostic skills (e.g., deliberating a differential diagnosis) but also topics in short- and long-term management, patient education, and socioeconomic determinants of health. By illustrating the treatments and patient education topics recommended by both the discussant and the actual treating physician, the course demonstrated for students the varying possible approaches to the same clinical problems.

Another strength of our course was the emphasis on aspects of longitudinal patient care, such as chronic care management, which is often neglected in case-based learning and other preclinical curricula. Both first- and second-year course participants reported a greater score change than the control group in knowing how clinicians work through cases on a step-by-step level in a longitudinal primary care setting, with a statistically significant difference found between first-year course participants and control students. Clinical and preclinical education typically provide more instruction in managing acute conditions with less time dedicated towards longitudinal care and chronic illness management. Due to an increasing population of patients with chronic conditions [49, 50] and studies suggesting physician dissatisfaction with their training in chronic illness care [51], medical educators have called for more instruction in longitudinal medical care and chronic disease management [51–54]. Via cases involving content over a median of 5 patient visits spanning over multiple months, our course involved nuanced discussions in managing conditions such as diabetes, hypertension, chronic kidney disease, and complications of cancer treatment. Increasing preclinical instruction in chronic illness management via case-based discussions is feasible and provides medical students with early exposure to this critical component of medical care.

### Limitations

A limitation of the blinded-discussant format of our curriculum is the potential pressure on discussants to arrive at the correct diagnosis. However, out of the three faculty that declined to participate as discussants (out of 15 invited), only one cited the blinded-discussant format as the reason for declining the invitation. This pedagogical method would need to be implemented at other institutions to evaluate the feasibility of involving faculty in this teaching format. The quasi-experimental design used to evaluate this curriculum also faces several limitations, including use of a non-validated assessment survey. Since clinical medical students at our institution are evaluated by skills in reporter-interpreter-manager-educator (RIME)-based competencies [34], we used the same scheme to evaluate our course. Although RIME-based tools have been developed and studied for the purpose of evaluating medical students, to the best of our knowledge, no similar RIME-based instrument exists for self-reported assessments of clinical skills. We developed our survey items based on literature on the RIME scheme [33, 55] and based on our institution’s RIME-centered evaluation method for clinical rotations [34]. Other limitations of our study design include the low survey response rate and possible selection bias since both the intervention and control groups voluntarily participated in the study, with survey respondents potentially being different than the non-respondents. It is possible that the results would have been different with randomized samples of students from our institution. Furthermore, we were unable to evaluate whether our curricular method led to long-term improvements in clinical skills among course participants compared with non-participants lasting beyond completion of medical school. Future studies evaluating this curricular format should compare objective measures of clinical skills between course participants and non-participants.

Further studies with larger sample sizes and a randomized design are needed to evaluate the effect of a faculty- and student-blinded pedagogical model on cognitive skills of students. This teaching model should also be attempted with smaller groups of students to evaluate whether faculty instructor blinding can be implemented towards traditional small-group case-based learning sessions.

## Conclusions

Case-based teaching continues to grow as an instrumental pedagogical model in preclinical education with an objective of imparting real-world clinical cognitive skills. We developed an elective curriculum that promoted prospective case-based discussions and avoided hindsight bias by having a blinded discussant and blinded group of students work through real case information in the chronologic sequence encountered by the treating physician. We piloted this course with intermediate-sized groups of students with significant improvements in self-assessed understanding of step-by-step clinical reasoning. Future attempts can be made to implement this format of two-way blinding of faculty and students in smaller-sized CBL sessions to further evaluate the feasibility and outcomes of prospective case-based teaching.

## Abbreviations

CBL: Case-based learning
CPC: Clinicopathologic conference
EMR: Electronic medical records
PCP: Primary care physician
RIME: Reporter-interpreter-manager-educator
